# Testing the reliability of hands and ears as biometrics: the importance of viewpoint

**DOI:** 10.1007/s00426-014-0625-x

**Published:** 2014-11-20

**Authors:** Sarah V. Stevenage, Catherine Walpole, Greg J. Neil, Sue M. Black

**Affiliations:** 1 Department of Psychology, University of Southampton, Highfield, Southampton, Hampshire SO17 1BJ UK; 2 Centre for Anatomy and Human Identification, College of Art, Science and Engineering, University of Dundee, Dundee, DD1 4EH Scotland, UK

## Abstract

Two experiments are presented to explore the limits when matching a sample to a suspect utilising the hand as a novel biometric. The results of Experiment 1 revealed that novice participants were able to match hands at above-chance levels as viewpoint changed. Notably, a moderate change in viewpoint had no notable effect, but a more substantial change in viewpoint affected performance significantly. Importantly, the impact of viewpoint when matching hands was smaller than that when matching ears in a control condition. This was consistent with the suggestion that the flexibility of the hand may have minimised the negative impact of a sub-optimal view. The results of Experiment 2 confirmed that training via a 10-min expert video was sufficient to reduce the impact of viewpoint in the most difficult case but not to remove it entirely. The implications of these results were discussed in terms of the theoretical importance of *function* when considering the canonical view and in terms of the applied value of the hand as a reliable biometric across viewing conditions.

**Electronic supplementary material:**

The online version of this article (doi:10.1007/s00426-014-0625-x) contains supplementary material, which is available to authorized users.

## Introduction

Whilst criminals have learned to hide their face, or disguise their voice, their hands may nevertheless provide an important biometric within a court setting (Delac & Grgic, 2004). Indeed, the visibility and identification of unique cues within the hand, such as vein patterns and skin features (Black, Mallett, Rynn & Duffield, 2009; Black, MacDonald-McMillan & Mallett, 2013; Black, MacDonald-McMillan, Rynn & Jackson, 2013; Jackson & Black, 2013), have been sufficient to support a number of recent criminal convictions. Alongside this, however, the inherent flexibility of the hand means that it may be viewed from a variety of different viewpoints and in a variety of different positions, potentially compromising its biometric value. The purpose of the present paper is to investigate the limits of the hand as a biometric cue through exploring the ability of viewers to match images as viewpoint changes.

Key in this enquiry is the concept of the ‘canonical view’. In their seminal paper, Palmer, Rosch and Chase (1981) found high agreement amongst participants in three tasks involving (1) rating the ‘goodness’ of an image of a familiar object, (2) forming a mental image of a familiar object and (3) selecting the best camera angle to take a photo of a familiar object. Importantly, high agreement resulted whether participants judged a limited set of views presented to them (Palmer et al., 1981), or generated their own views through unconstrained rotation of familiar objects in a real-time 3D virtual space (Blanz, Tarr & Bülthoff, 1999). The consistently preferred image was termed the ‘canonical view’ and Palmer et al. suggested that it provided a ‘privileged perspective’. Perhaps most importantly, Palmer et al. noted that the canonical view elicited faster responses in an object naming task (see also Bülthoff, Edelman & Tarr, 1995) and in a visual search task (Newell, Brown & Findlay, 2004). Moreover, Gomez, Shutter and Rouder (2008, Expt 2) demonstrated benefit of presenting the canonical image during a free-recall task extending the importance of canonicality from perceptual- to memory-based tasks. Indeed, when asked to recall the names of 171 objects encountered in a study list, participants were able to recall significantly more objects when studied from canonical images (41 %) than when studied from non-canonical images (33 %).^1^ When taken together, these studies implied a performance advantage when viewing canonical images, but a performance cost otherwise. Consequently, if a canonical view was also demonstrated for hands, then their reliability as a biometric may be thrown into question in situations in which the viewing conditions deviated from the canonical ideal.

### Attributes of the canonical image

Blanz et al., (1999) considered the attributes required to define a view as canonical. Three main characteristics were highlighted:

1. *Goodness of recognition,* through representing distinctive object characteristics and minimising occlusion,
2. *Familiarity*, through frequency of exposure, and
3. *Display of object functionality* through reflecting a characteristic mode of interaction.

For a novel object, the preferred or canonical view could only be based on the first of Blanz et al.’s criteria. Thus, a canonical view (if one existed) reflected only geometric aspects of the image itself, and agreement amongst viewers on the canonical view tended to be relatively low (see Cutzy & Edelman, 1994; Edelman & Bülthoff, 2002; Perrett & Harries, 1988). In contrast, for a familiar object, the canonical view could additionally be informed by *experience* (frequency of exposure to different viewpoints) and *understanding* (appreciation of function), and this tended to result in a greater consensus regarding the canonical view.

Laeng and Rouw (2001) offered support to suggest that the cardinal defining characteristic of the canonical view was its ‘frequency of exposure’. They reported that, whilst the canonical view of a familiar face was best represented by a ¾ profile (see also Troje & Bülthoff, 1996), the canonical view of one’s own face was closer to the frontal image, this being the view most frequently seen. However, it may be premature to define frequency of exposure as the most important aspect of canonicality. Indeed, the perspective from which we most often see an object may be inherently linked to the function that the object fulfils (the last of Blanz et al.’s criteria), and herein lies the basis for predictions for the current paper.

### The present study

Given the aim of exploring whether the hand, as a biometric, could be processed accurately across different views, the central question for the current paper was whether a canonical view existed for hands. If so, performance was expected to be optimal when presented with this canonical view, and was expected to be impaired when presented with a non-canonical view. This would be a damaging result when evaluating the hand as a biometric, as it would suggest that the processing of the hand would only be reliable under limited conditions. However, with canonicality potentially influenced by both frequency of exposure and object function, it may be anticipated that a flexible object such as a hand may frequently be observed from a variety of viewpoints and in a variety of positions as it carries out a range of functions (see Laeng, Carlesimo, Caltagirone, Capasso & Miceli, 2002). As such, it may be predicted that hands may not have as strong a preference for a single canonical view, and consequently may survive presentation across a range of views, compared to a more rigid object. To test this prediction, the processing of hand images was compared here to the processing of ear images. Both represent valuable biometric cues (see Yan & Boywer, 2007 for a review of ear recognition, and Black et al, 2009 for a review of hand recognition). However, the hand has a greater degree of flexibility and multifunctionality compared to the ear.

Performance was explored in a lab-based task designed to be analogous to that within a criminal investigation. Specifically, a traditional simultaneous matching task was used in which participants were asked to find the image (from 10 possibilities) that matched a target image. Given the preceding discussion, it was expected that both hand and ear processing may show sensitivity to a change in viewpoint, with optimal performance being associated with more optimal images. However, it was also expected that hands would be less affected by a change in viewpoint compared to ears because the non-rigidity of the hand provides for greater functionality and in turn, exposure to a larger array of viewpoints. As such, the present study is grounded in the predictions of canonicality across rigid and non-rigid cues, but provides an important test of the limits of the hand as a forensic biometric.

## Experiment 1: method

### Design

A 2 × 3 mixed design was used in which stimulus type (hands or ears) was varied between participants, and viewpoint (good, medium and poor) was varied within participants. Performance was tested by means of a ‘1 in 10’ task (Bruce et al., 1999) in which the participants’ task was to select one image (from an array of 10) that matched a target. Accuracy of performance was recorded.

### Participants

A total of 50 novice participants (35 females, 15 males) took part either on a volunteer basis or in return for course credit. Participants were randomly assigned to study either hands (*n* = 25, 18 females) or ears (*n* = 25, 17 females), and both the age range (*t*_(48)_ = 1.18, *ns*) and gender split ($\chi_{(1)}^{2}$ < 1, *ns*) were matched across the two groups. In addition, one hand expert and one ear expert provided baseline data for comparison purposes. Each gained their expertise through academic experience within the field of anatomy, with specialisation in the area of hands or ears to assist UK investigative processes either through the preparation of court evidence, or through facial reconstruction, respectively.

All participants reported normal, or corrected-to-normal, vision and did not recognise any individuals from either their hands or ears.

### Materials

#### Hand images

A bespoke set of stimuli was gathered from 42 individuals (20 females, 22 males) to provide two images of each of six viewpoints of the hand. The two images differed only in the direction of the light source, and hence in the pattern of shadows. Their collection ensured that the matching task involved two different images of the same hand. Consequently, reliance on simple picture-related cues in the matching task was minimised. The six viewpoints captured (1) the dorsal (back) surface of the hand laid flat, (2) the palmar surface of the hand laid flat, (3) the hand in a relaxed pose, (4) the hand viewed from above whilst holding a glass (5) the hand viewed from above whilst holding a pen, and finally (6) the hand viewed from above whilst holding a mobile phone. These six viewpoints were selected to capture a range of hand positions reflecting forensic ideals (dorsal and palmar views) and functional utility (grasping, writing, texting).

From this set, the images associated with 30 individuals were selected on the basis of a lack of distinguishing features such as pigmentation irregularities, tattoos, cuts or abrasions, nail irregularities, or significant levels of visible hair on wrists or knuckles. All individuals were photographed without jewellery and nail varnish.

#### Ear images

Ear images were obtained from the facial photographs of 116 individuals represented in the SuperIdentity Stimulus Database. The ears were extracted from full head images using Corel Photoshop such that the full extent of the ear was visible whilst minimising the amount of hair within the image. In this way, two ear images were extracted (for the reasons stated above) for each of six viewpoints capturing (1) the ear from the side, (2) the ear from a ¾ profile, and (3) the ear from the front as viewed both from a horizontal (0°) perspective and from a +20° perspective looking down. Again, these viewpoints were selected to reflect those available in optimal forensic contexts (mug-shots) and in more ecologically valid contexts such as from a closed-circuit television (CCTV) image where a camera is typically mounted above head height looking down.

From the set of images available, 30 individuals were selected to minimise visible head hair, and other distinguishing features such as lobe or helix irregularities, or multiple piercings. Again, all individuals were photographed without jewellery.

Both sets of stimuli were photographed using a Nikon D200 SLR camera under controlled artificial light conditions. The hands were photographed resting on a matt black horizontal surface, from a distance of approximately 45 cm. The (heads and) ears were photographed against an 18 % grey background from a distance of 1 m.

#### Determination of viewpoint quality

**Fig. 1:**
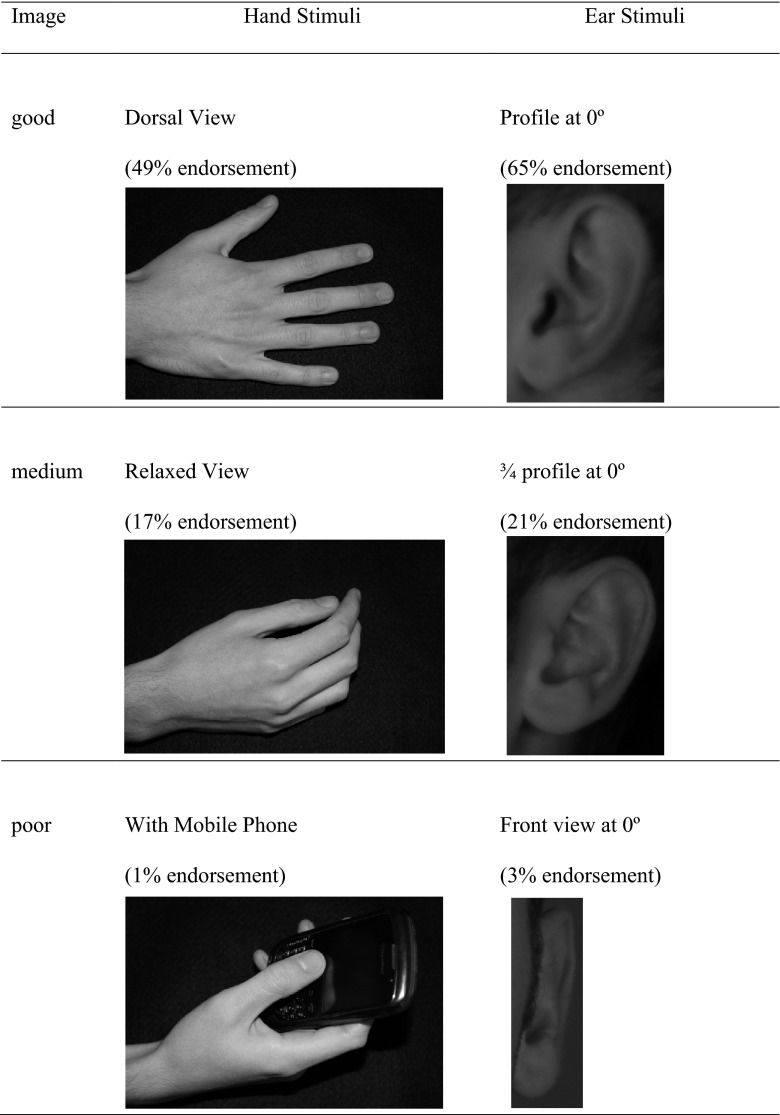
Example images depicting good, ‘medium and poor viewpoints for hands and for ears, together with their level of popularity (endorsement) across 100 individuals

To determine the quality of the viewpoints, a crowdsourcing technique (Mturk) was used in which 100 individuals were shown the 6 viewpoints for a single hand, and the 6 viewpoints for a single ear. In line with Palmer et al, (1981), their task was to select the image that best corresponded to the mental image that they formed in their mind’s eye when imagining a hand or an ear. For both hands and ears, the most popular viewpoint was nominated as the optimal or ‘good’ viewpoint. This was chosen by a minimum of 40 % of the individuals. Similarly, the viewpoint of intermediate popularity was nominated as the ‘medium’ viewpoint and this was chosen by approximately 20 % of the individuals. Finally, the viewpoint that was least popular was nominated as the non-optimal or ‘poor’ viewpoint, and was selected by less than 5 % of the individuals. Care was taken to balance the popularity of corresponding nominations across the hands and ears as far as possible. The resulting nominated viewpoints, and their level of popularity amongst the 100 individuals, are summarised in Fig. 1 and were used in the subsequent experimentation.

### Procedure

Across the experiment, participants completed 30 ‘1 in 10’ matching trials in which their task was to decide which, of a set of 10 images, matched the single target displayed simultaneously at the top of the computer screen. As such, this was a perceptual-matching task with no memory component and no naming requirement. All trials were ‘target present’ trials, however, the target image at the top of the screen and the image within the array were always two different images (even if in the same viewpoint) to prevent simple picture matching.

**Fig. 2:**
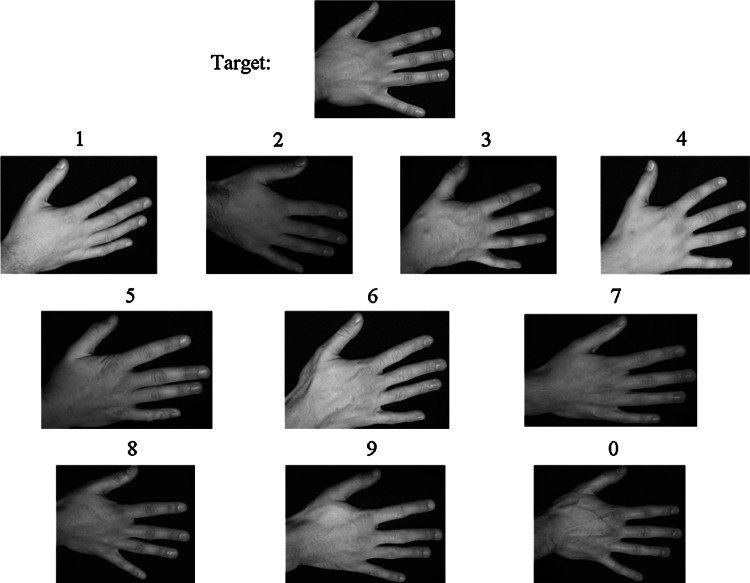
Example array for hands, with the target image depicted at the top of the display, and the 10 test images presented below. The target image was always depicted from the good viewpoint, whilst the test images were all depicted from either the good, medium or poor viewpoint. The target was always present amongst the test images but was always a different image. Here, the target is in position 8

The format of each trial was identical and consisted of the presentation of the target at the top of the screen, with the array of 10 images, in three rows of 4 (top), 3 (middle) and 3 (bottom), simultaneously displayed beneath it. Above each image in the array was a number to denote its position within the array, with positions 1–4 referring to locations from left to right on the top row, positions 5–7 referring to locations from left to right on the middle row, and positions 8–0 referring to locations from left to right on the bottom row (see Fig. 2).

The target image was always presented in the good viewpoint, analogous to the optimal image of a ‘suspect’s hand’ within an investigation. The array of 10 images all showed stimuli in either good, medium, or poor viewpoints with 10 trials for each viewpoint. These were blocked according to viewpoint. The order of these blocks, and the selection of individual target exemplars presented within each block, was counterbalanced across participants, to minimise the influences of fatigue and item effects within the study.

The participant’s task was to respond as quickly but as accurately as possible to indicate which of the 10 images in the array depicted the target at the top of the screen. Participants were aware that the image of the target in the array would be different and thus they were looking for a different image of the same hand (or ear) rather than an identical image. Participants indicated their answer by pressing the numbered key (0–9) on a standard keyboard that corresponded to the position of their selected image in the array, and all images remained visible until this response was made. Self-paced breaks separated the three blocks of trials and the entire experiment lasted approximately 30–40 min, after which participants were thanked and debriefed.

## Experiment 1: results and discussion

Accuracy on the ‘1 in 10’ task is summarised in Table 1 and was explored to determine whether novice performance on the matching task (1) was better than chance, (2) approached the level of the experts and (3) differed across viewpoint.

**Table 1:** Absolute and standardised accuracy of performance (and standard deviation) on the ‘1 in 10’ matching task for experts, novices (experiment 1) and trained participants (experiment 2)

	Good image	Medium image		Poor image
Hand recognition accuracy				
Expert (absolute)	1.00	0.90	0.33	
Novice (absolute)	0.52 (0.16)	0.45 (0.15)	0.23 (0.08)	
Trained (absolute)	0.53 (0.16)	0.44 (0.14)	0.21 (0.06)	
Novice (standardised)	1.00 (0)	0.89 (0.26)	0.50 (0.26)	
Trained (standardised)	1.00 (0)	0.88 (0.32)	0.43 (0.18)	
Ear recognition accuracy				
Expert (absolute)	0.87	0.70	0.40	
Novice (absolute)	0.63 (0.15)	0.27 (0.11)	0.17 (0.06)	
Trained (absolute)	0.54 (0.18)	0.32 (0.10)	0.19 (0.09)	
Novice (standardised)	1.00 (0)	0.44 (0.19)	0.29 (0.15)	
Trained (standardised)	1.00 (0)	0.65 (0.24)	0.39 (0.21)	

### Comparison to chance

To address the first question, a series of one-sample *t* tests was conducted comparing accuracy to a chance level of 0.1. These indicated that for both hands and ears, and across every viewpoint, novice participants were significantly better than chance (all *ts*_(24)_ > 5.93, *p* < 0.001). This was important in demonstrating the absence of floor effects within the data despite the very different nature of the hand and ear stimuli.

### Comparison to experts

To address the second question, one-sample *t* tests were conducted to compare the absolute performance of participants to that of the relevant expert at each viewpoint. As might be anticipated, these revealed that, whilst the novice participants performed at above chance levels, they performed below the level of the expert in all conditions (all *t*s_(24)_ > 7.63, *p* < 0.001).

### Impact of viewpoint

To address the final question, a 2 × 3 mixed Analysis of Variance (ANOVA) was conducted to explore accuracy of performance when matching hands and ears across good, medium and poor viewpoints. For this analysis, accuracy levels were standardised by expressing them as a proportion of the performance level attained in the optimal (good) condition (see Table 1). This ensured a focus on the relative impact of a *change* in viewpoint, and prevented the findings being affected by variation in absolute levels of performance across the stimuli.

The ANOVA revealed a main effect of stimulus type (*F*_(1, 48)_ = 41.59, *p* < 0.001, partial η^2^ = .464), with better overall performance for hands than for ears. In addition, a main effect of viewpoint emerged (*F*_(2, 96)_ = 409.52, *p* < 0.001, partial *η* ^2^ = 0.895), with better performance when presented with more optimal viewpoints. These effects were qualified by the expected interaction between stimulus type and viewpoint (*F*_(2,96)_ = 24.79, *p* < 0.001, partial *η* ^2^ = 0.34).

Analysis of the simple main effects confirmed a significant effect of viewpoint for both hands (*F*_(2,48)_ = 47.27, *p* < 0.001, partial *η* ^2^ = 0.66) and ears (*F*_(2,48)_ = 233.48, *p* < 0.001, partial *η* ^2^ = 0.907) suggesting that the performance for both stimulus types suffered as the view became less optimal. However, a series of Bonferroni-corrected comparisons confirmed that performance with hands was not affected by a change from good to medium images (*t*_(24)_ = 2.04, *p* > 0.05) but was only affected by a change from medium to poor images (*t*_(24)_ = 6.72, *p* < 0.001). In contrast, performance with ears was affected as soon as the image moved away from optimal, with significant differences in performance levels between good and medium images (*t*_(24)_ = 14.92, *p* < 0.001) as well as between the medium and poor images (*t*_(24)_ = 4.16, *p* < 0.001).

In accounting for these results, it was possible that ear processing was more affected by a change in viewpoint than hand processing because ear processing was an inherently difficult task. Important in this regard was the demonstration of equivalent absolute levels of performance in the best image case (*t*_(48)_ = 2.48, *ns*) despite the differences between hands and ears as stimuli. Consequently, the substantial impact of viewpoint for ears could not easily be attributed to an inherent difficulty when matching ears. However, the possibility remained that the difficulty when matching ears was revealed not in baseline performance levels, but in a greater vulnerability as the image quality was changed. Such an explanation was compatible with the predictions for this study in which the flexibility of the hand was expected to minimise the impact of a sub-optimal viewpoint. Indeed, these two accounts would be difficult to separate out.

Taking all analyses together, the results of Experiment 1 provided support for the predictions. Specifically, the change in viewpoint had a significant effect when matching hands, but had a greater effect, from an equivalent starting point, when matching ears. These results supported the prediction that the inherent flexibility of the hand-enabled exposure to a variety of viewpoints with the consequence that canonicality was less strong for hands than ears.

In terms of implications for the hand as a biometric, the data here led to the conclusion that when matching hands, performance could survive moderate changes in viewpoint whereas when matching other more rigid biometrics (such as ears), a change in viewpoint compromised performance quite substantially. As such, these data confirmed a greater reliability of the hand as a biometric cue across optimal and moderately optimal viewing conditions.

Several aspects of the current results were interesting and unanticipated, and as such warrant some consideration. In particular, it was interesting to note impairment in the performance of the two experts as viewpoint changed. Whilst it was not possible to assess the extent of the impact of viewpoint statistically for each of the experts (there being only one expert for each stimulus type), it was possible to determine whether the experts were affected to the same degree as the novice participants.

To this end, a series of one-sample *t* tests was conducted, comparing the decline in performance shown by the expert, to the decline in performance shown by the group of novices. This confirmed that novice performance declined more than expert performance as the viewpoint became less optimal. This was evident when matching ears as the image changed from good to medium (ears: *t*_(24)_ = 6.25, *p* < 0.001; hands: *t*_(24)_ = 1.08, *ns*), and when matching both ears and hands as the image changed from medium to poor (ears: *t*_(24)_ = 8.64, *p* < 0.001; hands: *t*_(24)_ = 11.23, *p* < 0.001). Consequently, these results suggested that whilst the experts were affected by a change in viewpoint, they were affected less than novices.

This latter analysis did not sit within the main purpose of this Experiment but nevertheless raised questions: For example, could the provision of training be sufficient to improve performance levels from that of the novice towards that of the expert. Relatedly, could the provision of training ameliorate the negative impact of the sub-optimal viewpoint so that trained participants come to show greater resilience than novices when presented with sub-optimal viewpoints?

Whilst representing an important applied issue, such questions relate well to the theoretical consideration of Blanz et al., (1999) regarding the criteria underpinning a canonical view. Indeed, it may be argued that expertise brings with it a capacity to use a range of cues so that the matching task can still be completed even when a subset of the cues is unavailable through occlusion in a sub-optimal image. Similarly, it may be argued that expertise brings the capacity to show better understanding of function, and greater levels of exposure to non-standard viewpoints through expert study. All factors may lead to the prediction that canonicality is less strong (or the negative impact of a non-canonical image can more easily be overcome) when the viewer brings expertise to their viewing task.

Experiment 2 was conducted to present an examination of these emergent questions. Through the provision of video instruction, the performance of a group of ‘trained’ participants was compared to that of the novices and experts studied in Experiment 1. It was anticipated that training would improve overall levels of performance, and would reduce the impact of a change in viewpoint compared to the novices such that the performance of the trained group would more closely resemble that of the experts.

## Experiment 2: method

### Design

The design was identical to that used in Experiment 1 except that training was provided via a short video prior to completing the ‘1 in 10’ trials. Accuracy on the matching task remained as the measure of performance.

### Participants

A total of 50 trained participants took part in return for a small monetary reward. Participants were randomly assigned to study either hands (*n* = 25, 16 females) or ears (*n* = 25, 14 females), and both the age range (*t*_(48)_ < 1, *ns*) and gender split ($\chi_{(1)}^{2}$ < 1, *ns*) were matched as before across the two groups.

### Materials

The ‘1 in 10’ materials were identical to those used in Experiment 1. In addition, however, two training videos were prepared. The videos lasted 12 min (hand training) and 11 min (ear training), and provided foundational input on the anatomy of the hand or ear, and the diagnostic features that would be examined by a forensic expert to determine a match between one sample and another for court purposes.

### Procedure

The procedure was identical to that in Experiment 1 with the exception that participants received video training on how to examine either hands or ears depending on the condition to which they had been assigned. The completion of the ‘1 in 10’ trials followed this training, and the entire task lasted up to 45 min, after which participants were thanked and debriefed.

## Experiment 2: results and discussion

Analysis within Experiment 2 took the same format as in Experiment 1 and results are summarised in Table 1. Performance in the ‘1 in 10’ task was examined to see whether it (1) was better than chance, (2) approached the level of the experts, and (3) differed across viewpoint.

### Comparison to chance

In terms of absolute performance levels, a series of one-sample *t* tests confirmed that performance for both hand and ear recognition across every viewpoint exceeded the chance level of 0.1 (all *ts*_(24)_ > 5.48, *p* < 0.001). This again demonstrated that there were no floor effects within the data.

### Comparison to experts

It was also evident that, whilst absolute levels of performance showed some improvement from novice levels, one-sample *t* tests still confirmed that the trained participants performed at a level below the experts in every condition (all *t*s_(24)_ > 9.37, *p* < 0.001). This may have reflected a lack of practice in the task itself despite training, as well as those ‘hard-to-articulate’ elements of expertise that the training video could not easily provide.

### Impact of Viewpoint

To explore the impact of viewpoint for the trained participants only, a 2 × 3 mixed ANOVA was conducted to examine the impact of stimulus (hand, ear) and viewpoint (good, medium, poor) on accuracy of performance. As in Experiment 1, this analysis was conducted using the standardised accuracy scores so that the relative impact of a *change* in viewpoint remained the focus. The results mirrored those from Experiment 1 in all respects. Specifically, a main effect of stimulus type emerged (*F*_(1, 48)_ = 4.83, *p* < 0.05, partial *η* ^2^ = 0.091) with performance being better for hands than for ears. In addition, a main effect of viewpoint emerged (*F*_(2, 96)_ = 160.10, *p* < 0.001, partial *η* ^2^ = 0.769) with better performance when presented with more optimal viewpoints. Finally, these effects were qualified by a significant interaction between stimulus type and viewpoint (*F*_(2, 96)_ = 7.14, *p* < 0.001, partial *η* ^2^ = 0.129).

Analysis of the simple main effects revealed a significant impact of viewpoint both when matching hands (*F*_(2, 48)_ = 69.50, *p* < 0.001, partial η^2^ = 0.743) and when matching ears (*F*_(2, 48)_ = 104.19, *p* < 0.001, partial η^2^ = 0.813). Moreover, as in Experiment 1, Bonferroni-corrected comparisons confirmed that performance with hands was not affected by a change from good to medium images (*t*_(24)_ = 1.88, *p* > 0.05) but was affected by a change from medium to poor images (*t*_(24)_ = 9.18, *p* < 0.001). In contrast, ear matching was impaired both when the images changed from good to medium (*t*_(24)_ = 7.47, *p* < 0.001) and when the images changed from medium to poor (*t*_(24)_ = 6.98, *p* < 0.001).

### Impact of training

Of most interest within the results was the question of whether training would improve performance in the matching task, and would ameliorate the effects of viewpoint noted in Experiment 1. To address this question, a 2 × 2 × 3 mixed ANOVA was performed on the standardised accuracy scores across Experiments 1 and 2, enabling examination of the effects of training (novice, trained), stimulus type (hands, ears), and viewpoint (good, medium, poor). The presence of the expected three-way interaction between all factors (*F*_(2, 192)_ = 3.17, *p* < 0.01, partial *η* ^2^ = 0.032) justified further exploration of the predictions through separate analyses for each stimulus type.

### Performance with hands

A 2 × 3 ANOVA was conducted to explore the effect of training (novice, trained) and viewpoint (good, medium, poor) when matching hands. Given that the expert showed an impairment as viewpoint became poorer, it was anticipated that the moderate effect of viewpoint revealed with novice participants in Experiment 1 may remain despite the training provided in Experiment 2. However, it was hoped that the magnitude of this effect may have reduced with training. In partial support of this expectation, the ANOVA revealed a significant effect of viewpoint (*F*_(2, 96)_ = 114.82, *p* < 0.001, partial *η* ^2^ = 0.705). However, there was no significant effect of training (*F*_(1, 48)_ < 1, *ns*). Unsurprisingly, therefore, no interaction emerged, confirming that the influence of viewpoint was not reduced by training (*F*_(2, 96)_ < 1, *ns*). Indeed, both the novice and trained groups showed the same pattern of performance, with ability remaining stable as the image quality reduced from good to medium (novice: *t*_(24)_ = 2.04, *ns;* trained: *t*_(24)_ = 1.88, *ns*), but showing a decline as the image quality reduced further from medium to poor (novice: *t*_(24)_ = 6.72, *p* < 0.001, trained: *t*_(24)_ = 9.18, *p* < 0.001).

### Performance with ears

A 2 × 3 ANOVA was conducted as above to explore the effect of training (novice, trained) and viewpoint (good, medium, poor) when matching ears. As above, it was anticipated that the effect of viewpoint noted with novices in Experiment 1 would remain, but that its magnitude may be reduced with training. Again, the ANOVA revealed the expected main effect of viewpoint (*F*_(2, 96)_ = 305.43, *p* < 0.001, partial *η* ^2^ = 0.86), confirming increasingly impaired performance as the image became poorer. In addition, and in contrast to the results described above, the main effect of training reached significance (*F*_(1, 48)_ = 9.85, *p* < 0.005, partial *η* ^2^ = 0.17) suggesting that participants performed significantly better with training than without. This was gratifying to see as it confirmed the value of the training video for the participants working with the most vulnerable stimulus set. Most importantly, however, the anticipated interaction between training and viewpoint reached significance (*F*_(2, 96)_ = 7.23, *p* < 0.001, partial *η* ^2^ = 0.131).

Post hoc contrasts confirmed that performance fell significantly for both novice and trained groups as the image quality fell from good to medium (novice: *t*_(24)_ = 14.92, *p* < 0.001; trained: *t*_(24)_ = 7.47, *p* < 0.001), and as it fell further from medium to poor (novice: *t*_(24)_ = 4.16, *p* < 0.001; trained: *t*_(24)_ = 6.98, *p* < 0.001). However, the performance of the trained group was affected less (35 %) than that of the novice group (56 %) as the image quality reduced from good to medium (*t*_(48)_ = 3.45, *p* < 0.001). Consequently, and in line with predictions, the data confirmed that training significantly minimised the negative impact of the sub-optimal image.

Experiment 2 was conducted to determine whether training through simple instruction would increase performance levels from those displayed by the novices in Experiment 1, and would accordingly reduce the impact of a sub-optimal viewpoint. The results in this regard are equivocal. Training only had a significant effect on performance levels when matching ears. As a consequence, these trained participants did indeed show less impact of the sub-optimal viewpoint compared to the novice participants in Experiment 1. In this regard, training achieved its predicted purpose, whilst not raising performance levels up to those of the expert and whilst not removing the viewpoint effect altogether.

In contrast, and somewhat disappointingly, training had no significant effect on performance when matching hands. Consequently, it was unsurprising that that the viewpoint effects noted with novices in Experiment 1 remained evident for trained participants in Experiment 2. Notwithstanding this, it is worth noting that when matching hands, both novices and trained participants showed no significant decline in performance as the image quality fell from good to medium, and only showed a significant decline in performance as the image quality fell to an unacceptably poor level.

In reflecting on the lack of effectiveness of the hand training video, we can find no clear and satisfactory explanation. We considered, for example, the possibility that the video training was ineffective because it was unable to capture those heuristic expert strategies that may elude conscious awareness or clear articulation. This, by definition, remains likely, although it is difficult to see how this might apply to the hand training video but not to the ear training video. Hence, this remains unsatisfactory as an explanation of the current pattern of results.

We considered, also, the possibility that the training video for hands merely formalised the approach that the novices intuitively used and thus provided no additional benefit. Indeed, the demonstration of stable performance across novices and trained participants even in the best of viewpoint conditions might lend weight to this as an explanation. Our review of the video training suggests that, whilst possible, this may be unlikely as an explanation. The hand training concentrated on noticing the existence of one hand characteristic *relative* to another (i.e., the position of skin features relative to morphological characteristics such as knuckle creases). In comparison, the novice hand participants in Experiment 1 tended to comment on isolated hand features only. Consequently, whilst possible, it seems unlikely that ineffectiveness of the training video was due to it merely formalising the intuitive strategies of the hand novices.

What was clear, however, was that the participants in the hand-matching task performed at an equivalent level to those in the ear-matching task and performed some way below a ceiling level of performance. Consequently, we can reject a simple explanation in terms of a lower *capacity* for those in the hand-matching condition to improve with training.

In conclusion, the results of Experiment 2 suggested that the capacity to match hands was not improved by training, with the consequence that small changes in viewpoint were tolerated but larger changes in viewpoint still compromised performance. However, training was effective for participants when matching ears, and as a consequence, the negative impact of moderate viewpoint changes was significantly reduced, though not removed entirely.

## General discussion

The purpose of the present paper was to provide an empirical test of the reliability of the hand as a biometric cue when matching a sample to a suspect. The particular question being asked was whether this matching task could still be performed to an adequate level when the viewpoint of the hand changed from optimal, to sub-optimal. Performance here was assessed across a moderate change in viewpoint and across a substantial change in viewpoint. In addition, performance was assessed relative to a control condition in which ears represented the biometric cue. This combination of conditions allowed a test of the prediction that the hand, as an inherently flexible biometric cue, would better survive a change to a sub-optimal viewpoint compared to the ear as a rigid biometric cue.

The results of Experiment 1 confirmed the predictions in all respects. Whilst the matching of both hands and ears was affected by viewpoint changes, hands were affected to a lesser extent. Indeed, no significant decline was observed in hand-matching performance when the viewpoint change was moderate, and performance only significantly declined when the viewpoint change was substantial to provide an unacceptably poor image. In contrast, performance significantly declined when matching ears as soon as any deviation from the ideal viewpoint was introduced. The results of Experiment 2 revealed that simple training minimised these effects for ears when moderate viewpoint changes were introduced, but could not remove the negative effects of viewpoint altogether.

Importantly, these results now provide demonstration of the limits under which the matching of hand images can be considered stable and reliable. As a relatively new biometric, these results are important for the forensic community. Furthermore, they assume particular relevance given the recent concerns over susceptibility to bias amongst forensic scientists in exactly these sorts of matching tasks (see guidance report by the Forensic Science Regulator, 2014; commissioned report by the National Academy of Sciences (NAS), 2009). Both reports note the bias that can arise in decision making when conclusions are based on expert interpretation rather than scientific or metric analysis. Moreover, both reports note that such biases are ‘common features of decision-making and cannot be willed away’ (NAS, 2009, pp 122). For example, when making a decision on whether a latent fingerprint was a match to a suspect, expert interpretation was demonstrably affected by the presentation of fictitious contextual details to bias the outcome one way or the other (Dror & Charlton, 2006). Similar evidence exists in the arenas of DNA matching (Dror & Hampikian, 2011), and more recently in connection with forensic anthropology judgements of sex, ancestry and age at death (Nakhaeizadeh, Dror & Morgan, 2014). In light of such concerns over forensic science judgements through context or framing effects, the importance of research to document the limits of forensic interpretation is noted. Here, the demonstration of reliability when matching hands despite changes in viewpoint goes some way to defining the value of hand matching in a legal setting.

Having said this, it is important to note that whilst levels of performance when matching hands were not significantly affected by moderate changes to viewpoint, those levels of performance demonstrated by both novices and trained participants, were not high. In this regard, it is worth reflecting on the performance of the two experts who provided baseline data within Experiment 1.

Both experts were affected by a change in viewpoint, showing a small decline in performance with a moderate change in viewpoint, and showing a more substantial decline in performance with a more significant change in viewpoint. Interestingly, their confidence dropped sharply when presented with poor images, and in this sense, the experts showed a good metacognitive awareness that their performance had been severely compromised. Confidence from the novices and trained participants suggested less awareness than the experts of their compromised performance.^2^ Consequently, one important difference between the experts and the participants here is not necessarily in their ability but in their awareness of their ability. This metacognitive monitoring represents an area of emergent interest in the forensic field not least because of its potential to indicate when someone has sufficient belief in their ability to report their testimony in a formal context (see Brewer, 2006 for a useful review). However, to establish forensic value, courts will have to establish the confidence levels that they deem acceptable for the purposes of evidential admissibility.

The current study has been heavily influenced by the applied question of whether the hand remains of value as a biometric cue despite changing viewing conditions. However, the work described here is also grounded in a well-established literature regarding canonicality. In this regard, the work presented here may usefully contribute to discussions regarding the cardinal and defining characteristics of the canonical image. From the early work of Blanz et al., (1999), the defining attributes of the canonical image were identified as recognisability, frequency of exposure, and display of functionality. Whilst previous work had placed importance on frequency of exposure, the current results offer a challenge to this. In fact, one may consider that the display of functionality may be the most important aspect of a canonical image in that it may influence both additional attributes. More specifically, functionality is likely to determine those distinctive aspects of an object that must be portrayed if the object is to be recognisable. Similarly, functionality is likely to influence that view of an object that is most often seen. When presented with a multifunctional or non-rigid object capable of changing shape to fulfil several functions, what is clear is that several defining characteristics will make for a recognisable image, and similarly, several viewpoints will make for a good display of (at least one) function and are likely to drive frequency of exposure. The concept of a single canonical image consequently breaks down and, as seen here, performance on a simple perceptual task can remain robust across viewpoints.

This perspective sits well with more recent discussions regarding the importance of function in canonicality. Specifically, Woods, Moore and Newell (2008) demonstrated the novel concept of haptic canonicality—a preferential view from which an object may be identified by touch. Their participants showed substantial consistency when orienting an object to ‘an optimal position for learning by touch’. Moreover, these canonical haptic views did indeed lead to better haptic recognition. This is supported by two observations noted within the literature. First, when imaging a familiar asymmetric object such as a teapot, right-handed participants tended to place the handle on the right as they would when grasping it (Blanz et al., 1999, Expt 2). Second, and more interesting, cases of agnosic patients have been documented who showed an inability to recognise an object from the retinal image, but could instantly identify the object when permitted to pick it up and handle it (see Riddoch & Humpheys, 1987).

Together, these findings support our demonstration that canonicality may depend rather critically on the display of functionality rather than just frequency of exposure. The implication tested here was that multifunctional objects would show weaker preference for a single canonical view, and greater tolerance of changes in view. Within the forensic arena where the matching of biometric cues may be of interest, these results hold value in defining the limits under which performance may remain reliable. However, within a more theoretical arena, these results also hold value as we refine our understanding of the canonical view.

## Electronic supplementary material

Below is the link to the electronic supplementary material.

Supplementary material 1 (DOCX 20 kb)

## Footnotes

1 It should be noted the Gomez et al. (2008, Expt 1) failed to show a clear advantage of canonical study images when participants were required to recognise objects. When conditions permitting picture-related processing are excluded, the novelty of the non-canonical image actually triggered more hits and fewer false alarms than the canonical image.

2 Whilst it was not a main focus of the current paper, confidence ratings were collected as is standard in the ‘1 in 10’ task. For an analysis of the confidence ratings within this study, please refer to supplemental materials.
