# Supplementary material for: Testing the reliability of hands and ears as biometrics: the importance of viewpoint
Source: Psychol Res. 2014 Nov 20;79(6):989–99. doi: 10.1007/s00426-014-0625-x (PMC4624835; doi:10.1007/s00426-014-0625-x)
Supplement: Supplementary file 1 — Supplementary material 1 (DOCX 20 kb) [file 426_2014_625_MOESM1_ESM.docx]

Supplemental Materials: Analysis of Confidence

Whilst not the primary focus of the current paper, self-ratings of confidence in decision making were recorded within the ‘1 in 10’ task, and analyses are provided here for completeness.

Experiment 1:

Within Experiment 1, performance when matching hands and ears in the 1 in 10 task was recorded across three viewpoints: good, medium and poor. Accuracy of performance was recorded and is reported in the main paper. Self-reported confidence was also gathered, through participants ratings on a 7 point scale to indicate how confident they felt in their decision for each trial (1 = not at all confident; 7 = very confident indeed). These are reported in Table 1.

(Please insert Table 1 about here)

The Analysis of confidence ratings revealed a very similar picture to the analysis of accuracy. Analyses were conducted to determine whether confidence (i) differed across viewpoint and (ii) approached the level of the experts.

*Impact of Viewpoint:* A 2 x 3 mixed ANOVA was conducted in order to address the impact of viewpoint on confidence when recognising hands and ears. This confirmed that novice participants were more confident when matching hands than ears (*F*_(1, 48)_ = 5.26, *p* < .05, partial η^2^ = .97), and when presented with good images rather than less optimal images (*F*_(2, 96)_ = 126.22, *p* < .001, partial η^2^ = .72). These effects were qualified by the expected interaction between stimulus and viewpoint (*F*_(2, 96)_ = 10.09, *p* < .001, partial η^2^ = .17).

Analysis of the simple main effects confirmed a main effect of viewpoint on confidence both for hand matching (*F*_(2, 48)_ = 32.25, *p*< .001, partial η^2^ = .57) and ear matching (*F*_(2, 48)_ = 108.82, *p* < .001, partial η^2^ = .82) suggesting that confidence suffered as the image became worse. As with the accuracy analysis, however, the magnitude of the effect was smaller when matching hands (*t*_(48)_ = 3.73, p < .001) confirming less of a decline in confidence when processing hands than ears across viewpoints.

*Comparison to Experts:* Comparison of confidence levels between our novice participants and our experts via a series of one-sample *t*-tests suggested some differences between the two groups. Novice participants showed equivalent levels of confidence when matching hands from the good images (*t*_(48)_ < 1, *ns*) and the medium images (*t*_(48)_ = 1.25, *ns*) but had more confidence in their decisions relative to the expert when images were poor (*t*_(48)_ = 11.49, *p* < .001). In contrast, whilst less confident in their performance when images were good *t*_(48)_ = 2.86, *p* < .01), those participants involving in matching ears had equivalent levels of confidence to the experts only when images were medium (*t*_(48)_ = 1.06, *ns)*. As above, they were significantly more confident than the experts when ear images were poor (*t*_(48)_ = 3.26, *p* < .005).

Taken together, these results suggest that the experts realised the impact that a poor image may have on their ability, and their confidence was lower as a result. However, the novice participants did not adjust their confidence levels accordingly and were more confident relative to the experts in the hardest condition despite the fact that their performance was significantly worse.

Experiment 2

Within Experiment 2, training was introduced through the presentation of an 11-12 minute instructional video. This provided otherwise-naive participants with foundational information of relevance when matching either hand images or ear images. As such, Experiment 2 sought to determine whether performance after training would improve from the novice baseline, would approach the level of the experts, and would still suffer when viewpoint was less than optimal.

As in Experiment 1, accuracy on the 1 in 10 matching task was recorded, and represented the focus of interest. Analyses are reported in the main paper. However, again, self-rated confidence was collected through participants ratings on a 7 point scale to indicate how confident they felt in their decision for each trial (please see Table 1).

The analysis of confidence revealed a similar picture to accuracy within Experiment 2, and again explored whether confidence (i) approached the level of the experts, (ii) had increased as a result of the training, and (iii) still differed according to viewpoint.

*Comparison to Experts*: In order to address the first question, a series of Bonferoni-corrected one-sample *t*-tests compared the confidence of the trained participants to that of the expert in each condition. When recognising hands, the trained participants showed equivalent levels of confidence to the expert when recognising from ‘good’ images (*t*(24) = 2.26, *ns*) and from ‘medium’ images (*t*(24) < 1, *ns*). However, they were more confident than the expert when recognising from the ‘poor’ images despite the fact that their performance was significantly worse (*t*(24) = 7.21, *p* < .001). These results mirrored those in Experiment 1 in all respects.

In a similar vein, analysis of confidence in ear matching confirmed that the trained participants in Experiment 2 had equivalent levels of confidence to the expert when recognising from ‘good’ images (*t*(24) = 1.04, *ns*), and from ‘medium’ images (*t*(24) = 1.27, *ns*), but they remained more confident than the expert when matching from the ‘poor’ images despite their worse performance (*t*(24) = 4.40, *p* < .001). As such, confidence in ear matching following training showed the same pattern as confidence in hand matching, deviating slightly in this regard from the results of Experiment 1.

*Impact of Training and Viewpoint:* In order to see whether training had made a difference to self-rated confidence, and whether viewpoint effects still existed, a 2 x 2 x 3 mixed ANOVA was conducted, with training, stimulus type, and viewpoint as the independent variables. Surprisingly, this revealed no overall influence of training, either alone (*F*_(1, 96)_ < 1, *ns*) or in combination with any other variable (all *F*s < 2.84, *p* > .05, *ns*). The effect of viewpoint, however, remained strong and significant (*F*_(2, 192)_ = 242.03, *p* < .001, partial η^2^ = .716) with confidence falling as the viewpoint became worse.

Only one interaction emerged to qualify this result and this was the expected interaction between stimulus type and viewpoint (*F*_(2, 192)_ = 24.12, *p* < .001, partial η^2^ = .20). The emergence of this interaction echoed the results of Experiment 1. As before, analysis of the simple main effects confirmed a main effect of viewpoint on confidence for both hand matching (*F*_(2, 98)_ = 60.78, *p* < .001, partial η^2^ = .554) and ear matching (*F*_(2, 98)_ = 210.44, *p* < .001, partial η^2^ = .811) suggesting that confidence suffered in both cases as the image became worse. However, the magnitude of the effect was smaller for hand matching (*t*_(98)_ = 5.93, p < .001) confirming less of a decline in confidence when matching hands than ears across viewpoints, despite training.

Table 1:

Self-Rated confidence (with Standard Deviation) for experts, novice and trained participants in the ‘1 in 10’ task when matching hands and ears across different quality images.

|  | ‘Good’ Image | ‘Medium’ Image | | ‘Poor’ Image |
| --- | --- | --- | --- | --- |
| Hand Recognition |  |  |  | |
| Expert Confidence  Novice Confidence  Trained Confidence | 5.07  5.03 (.97)  4.52 (1.25) | 4.26  4.52 (1.04)  4.41 (1.10) | 1.33  3.51 (.95)  3.23 (1.32) | |
| Ear Recognition |  |  |  | |
| Expert Confidence  Novice Confidence  Trained Confidence | 5.63  5.18 (.79)  5.40 (1.11) | 4.03  3.8 (.93)  4.32 (1.12) | 1.8  2.5 (1.07)  2.75 (1.08) | |
